# Is AD a Stress-Related Disorder? Focus on the HPA Axis and Its Promising Therapeutic Targets

**DOI:** 10.3389/fnagi.2019.00269

**Published:** 2019-09-27

**Authors:** Geoffrey Canet, Célia Hernandez, Charleine Zussy, Nathalie Chevallier, Catherine Desrumaux, Laurent Givalois

**Affiliations:** Molecular Mechanisms in Neurodegenerative Dementia Laboratory (MMDN), INSERM, U1198, Environmental Impact in Alzheimer’s Disease and Related Disorders (EiAlz) Team, EPHE, University of Montpellier, Paris, France

**Keywords:** Alzheimer’s disease, glucococorticoids, stress-related disorder, CRH (corticotropin-releasing hormone), HPA axis (hypothalamus-pituitary-adrenal), AVP (arginine vasopressin), 11β hydroxysteroid dehydrogenase

## Abstract

Alzheimer’s disease (AD) is a progressive neurodegenerative disorder that has important health and economic impacts in the elderly. Despite a better understanding of the molecular mechanisms leading to the appearance of major pathological hallmarks (*senile plaques and neurofibrillary tangles*), effective treatments are still lacking. Sporadic AD forms (*98% of all cases*) are multifactorial, and a panoply of risk factors have been identified. While the major risk factor is aging, growing evidence suggests that chronic stress or stress-related disorders increase the probability to develop AD. An early dysregulation of the hypothalamic-pituitary-adrenal axis (HPA axis or stress axis) has been observed in patients. The direct consequence of such perturbation is an oversecretion of glucocorticoids (GC) associated with an impairment of its receptors (glucocorticoid receptors, GR). These steroids hormones easily penetrate the brain and act in synergy with excitatory amino acids. An overexposure could be highly toxic in limbic structures (*prefrontal cortex and hippocampus*) and contribute in the cognitive decline occurring in AD. GC and GR dysregulations seem to be involved in lots of functions disturbed in AD and a vicious cycle appears, where AD induces HPA axis dysregulation, which in turn potentiates the pathology. This review article presents some preclinical and clinical studies focusing on the HPA axis hormones and their receptors to fight AD. Due to its primordial role in the maintenance of homeostasis, the HPA axis appears as a key-actor in the etiology of AD and a prime target to tackle AD by offering multiple angles of action.

## General Aspects

Sporadic Alzheimer’s disease (AD; *98% cases*) is a progressive neurodegenerative pathology, and its complexity could be explained by a wide range of risk factors. Growing evidence suggests that lifetime events such as chronic stress or stress-related disorders, like major depression disorder (MDD) or anxiety, may increase the probability to develop AD (Heininger, 2000; Blennow et al., 2006; Querfurth and LaFerla, 2010; Canet et al., 2019). In patients, an early dysregulation of the hypothalamic-pituitary-adrenal (HPA) axis was observed (Hartmann et al., 1997; Csernansky et al., 2006). The direct consequence of such perturbation is a glucocorticoids (GC) over-secretion associated with GC receptors (GR) signaling impairment. These steroid hormones easily penetrate the brain tissue and act in synergy with excitatory amino acids. A GC overexposure is highly toxic in limbic structures (McEwen, 2008), especially in prefrontal cortex and hippocampus, which could participate to the cognitive decline occurring in AD. Furthermore, GC and GR dysregulations are involved in lots of functions disturbed in AD: i.e., dysregulation of the amyloid precursor protein (APP) processing, Tau phosphorylation, neuroinflammation, oxidative stress and excitotoxicity (Sapolsky, 1996; McEwen, 2008; Bengoetxea et al., 2016). Thus, a vicious cycle between AD and the HPA axis seems to occur, where AD induces the dysregulation of the HPA axis, which in turn potentiates the pathology (Brureau et al., 2013; Pineau et al., 2016; Canet et al., 2019).

This article reviews some preclinical and clinical studies focusing on the HPA axis hormones and receptors as potential targets for AD. Promising results are obtained by targeting corticotropin releasing hormone (CRH), arginine vasopressin (AVP) and GR, or by inhibiting the 11β-hydroxysteroid dehydrogenase-1 (11β-HSD1), involved in GC synthesis. Finally, this review intends to show that the HPA axis, due to its essential role in the maintenance of homeostasis, could be a key factor in the etiology of AD and a prime target to tackle AD by offering multiple druggable opportunities.

## The HPA Axis

**Figure 1:**
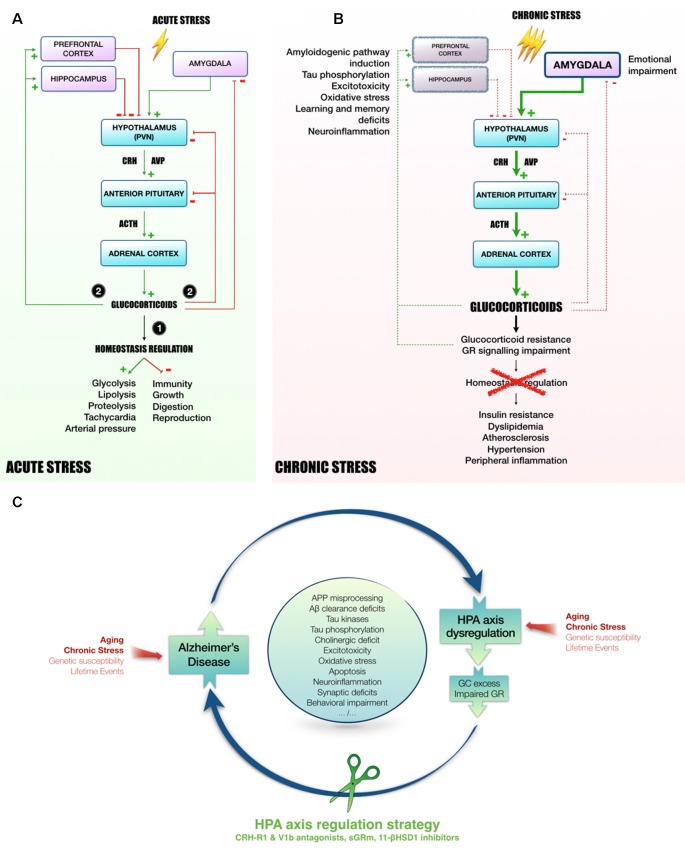
Mechanisms linking HPA axis dysregulation and AD. Following acute stress **(A)**, hypothalamic PVN releases CRH and AVP in the blood portal of median eminence. In response to CRH and AVP, corticotropic cells of anterior pituitary release ACTH in the peripheral circulation to induce GC secretion in blood by adrenal cortex. Succinctly, (1) GC mobilize energy resources and increase cardiovascular function to fight stress. Besides, GC inhibit unnecessary functions in the early phase of stress response, such as immunity, growth, digestion and reproduction. Then, (2) to avoid runaway of the system, GC exert an inhibitory feedback at all stages of HPA axis (hypothalamus and pituitary). In addition, as they easily penetrate in the brain, GC also act on several regions involved in the control of HPA axis activity, such as hippocampus and prefrontal cortex (*tonic inhibition*) or amygdala (*tonic stimulation*, Canet et al., 2018). However, chronic stress leads to a sustained activation of HPA axis and could induce stress-related disorders, as for instance MMD and AD (**B**, Canet et al., 2018). In this context, GC over-secretion is associated with GC resistance and GR signaling impairment (Chrousos et al., 1993). Homeostasis maintenance is compromised, leading to insulin resistance, dyslipidemia, atherosclerosis, hypertension and a massive peripheral inflammation (Vitellius et al., 2018; Maslov et al., 2019). In limbic structures (*hippocampus, prefrontal cortex*), it was shown that GC overexposure induces hippocampal and cortical atrophy (McEwen, 2008) and amygdala hypertrophy (Vyas et al., 2003, 2004), that could be related to learning and memory deficits, emotional impairment, excitotoxicity, neuroinflammation and oxidative stress (Sapolsky, 1996; McEwen, 2008; Bengoetxea et al., 2016). In the AD context, high levels of GC, and the dysregulation of the HPA axis activity observed in patients (Hartmann et al., 1997; Swanwick et al., 1998), seems to be particularly involved in the induction of amyloidogenic pathway and the abnormal phosphorylation of Tau (Green et al., 2006; Pineau et al., 2016; Sotiropoulos and Sousa, 2016; Vyas et al., 2016; Canet et al., 2019). Thus, it appears that the rise of circulating GC increases AD pathology, resulting in a vicious cycle by which pathology induces HPA axis dysregulation, GC overexposure and GR signaling impairment, which in turn potentiates the pathology. Due to its primordial role in the maintenance of homeostasis, targeting HPA axis offers multiple angles of action to break this vicious cycle and pave the way to new therapeutic strategies **(C)**. Abbreviations: 11-βHSD1, 11β-hydroxysteroid dehydrogenase-1; Aβ, amyloid-β protein; APP, amyloid precursor protein; ACTH, adrenocorticotropin; AD, Alzheimer’s disease; AVP, arginine-vasopressin; CRH, corticotropin releasing hormone; CRH-R1, CRH receptor type 1; GC, glucocorticoids; GR, glucocorticoid receptors; HPA axis, Hypothalamic-pituitary adrenal axis; MDD, Major depressive disorder; PVN, paraventricular nucleus; sGRm, Selective GR modulator; V1b, Arginine-vasopressin receptor sub-type 1b.

The HPA axis is required to provide appropriate adaptation to external or internal challenges called stress, which initiates a cascade of hormonal processes (Figure 1A). This cascade starts in the hypothalamic paraventricular nucleus (PVN) with a release of CRH and AVP at the median eminence level. AVP acts in synergy with CRH to induce pituitary adrenocorticotropin (ACTH) release in blood (Whitnall et al., 1987; Mouri et al., 1993; Raff, 1993; Torner et al., 2017). Then, ACTH triggers the adrenal cortex to release GC (cortisol in human, corticosterone in rodents). These hormones act widely throughout the body and brain to mobilize energy resources in order to fight stress and maintain homeostasis (Carroll et al., 2011). To avoid an overactivation of the HPA axis, GC exert an inhibitory feedback at all stages of the axis (Figure 1A; Tasker and Herman, 2011). These steroid hormones bind to low affinity GR and high affinity mineralocorticoid receptors (MR; Reul and de Kloet, 1985). These nuclear receptors are necessary for normal cellular activity and crucial for many central nervous system functions, including learning and memory (Roozendaal, 2000). The GC circadian regulation is under the control of MR, while under stress conditions, GC rise significantly causing substantial activation of GR (Reul and de Kloet, 1985; Thomas, 2015).

It is interesting to note that AVP and CRH are also locally synthesized within the brain, where they are important neuromodulators involved in the central organization of various brain-mediated stress responses (Buijs, 1990; Tilders et al., 1993).

In human, chronic stress and HPA axis dysregulation with chronic GC hypersecretion appear to exert detrimental effects in normal aging and AD (Hartmann et al., 1997; Lupien et al., 1998; Notarianni, 2013; Givalois, 2014; Canet et al., 2018), but also in Parkinson’s disease (Wu et al., 2016), Cushing’s syndrome (Kroon et al., 2018), and MDD (Canet et al., 2018). A prolonged exposure to GC appears to damage particularly limbic structures (*hippocampus and prefrontal cortex*) by inducing a state of vulnerability in these neurons through the disruption of various cellular mechanisms (Sapolsky, 1996; McEwen, 2008). This review will present several current strategies in AD, aiming to restore HPA axis function and to limit GC production and toxicity.

## HPA Axis: Multiple Promising Targets for AD Treatment

### Targeting CRH Receptors In AD

CRH acts *via* G-protein-coupled receptor type 1 or 2 (CRH-R1 and CRH-R2) with widespread brain expression to orchestrate the stress response (Bale and Vale, 2004). In AD patients, investigators reported a reduction of immunoreactive cells for CRH in the cortex, associated with an increase of postsynaptic CRH receptors density (Whitehouse et al., 1987; Pomara et al., 1989; Behan et al., 1995). Besides clinical evidence, it was shown that CRH and CRH-R1 play critical roles in the regulation of stress-induced neuropathogenesis and behavioral deficits in mice models of AD (Dong et al., 2008; Carroll et al., 2011; Zhang et al., 2016; Table 1). CRH overexpressing mice display increased Tau hyperphosphorylation and aggregation in the hippocampus (Campbell et al., 2014). In Tg2576-AD mice, chronic isolation stress increases the expression of CRH-R1 in cortex and hippocampus (Dong et al., 2008). These authors also showed that chronic administration of antalarmin (*a CRH-R1 antagonist*) significantly decreased plasma corticosterone levels, tissue Aβ_1–42_ levels and Aβ plaques deposition in the brain, and blocked the effects of isolation stress on anxiety levels and memory (Dong et al., 2014). A few years later, a 3 month-treatment in food with CRH-R1 antagonists (*Antalarmin and R121919*) was shown to prevent stress-induced behavioral changes (*anxiety and memory*) and synaptic loss in aged rats, perhaps by reversing HPA axis dysfunction (Dong et al., 2018). Moreover, in another mouse model of AD (*APP/PS1*) a 5 month-treatment with R121919 prevented the onset of cognitive impairment, reduced cellular and synaptic deficits and Aβ levels (Zhang et al., 2016). Similarly, pre-treatment with another CRH-R1 antagonist (*NBI27914*) in PS19-AD mice (*P301S mutation*) before restraint/isolation stress prevented Tau hyperphosphorylation and aggregation, neurodegeneration and fear-memory impairment (Carroll et al., 2011).

**Table 1:** Preclinical and clinical studies targeting corticotropin releasing hormone (CRH), arginine vasopressin (AVP), glucocorticoids (GC) or glucocorticoid receptors (GR).

	Context/model	Molecular, cellular & behavioral impacts	Reference
**CRH receptors inhibition**			
CRH overexpression	C57/B16 mice	Increase Tau phosphorylation and aggregation.	Campbell et al. (2014)
Antalarmin and R121919 (CRH-R1 antagonists)	Aged rats	Prevention of stress-induced memory deficits and anxiety; Prevention of stress-induced synapse loss and HPA axis dysfunction.	Dong et al. (2018)
R121919 (CRH-R1 antagonist)	APP/PS1 mice	Prevention of the onset of cognitive impairment; Reduction of cellular and synaptic deficits; Decrease of Aβ and C-terminal fragment levels.	Zhang et al. (2016)
NBI 27914 (CRH-R1 antagonist)	PS19 mice	Prevention of stress-induced Tau hyperphosphorylation and aggregation, neurodegeneration and fear memory impairment.	Carroll et al. (2011)
Antalarmin (CRH-R1 antagonist)	Tg2576-AD mice	Decrease level of plasma Aβ_1–42_ and Aβ plaque deposits; Decrease level of plasma corticosterone; Improve memory and anxiety behavior.	Dong et al. (2014)
	Primary hippocampal culture	Inhibition of Aβ_1–42_ levels and PKA expression after a CRH treatment.	
**AVP receptors inhibition**			
SSR149415 (V1b antagonist)	Anxiety/depression rodent models	Anxiolytic-like activity in models involving traumatic stress exposure; Antidepressant-like effects in FST.	Griebel et al. (2002)
TASP0390325 and TASP0233278 (VI b antagonists)	Depression rodent models	Antidepressant-like effects in the FST; Reduction of the hyperemotionality after olfactory bulbectomy.	Iijima et al. (2014)
ABT-436 (V1b antagonist)	Human (MDD subjects)	Phase 1b in clinical trial for MDD; Reduction of HPA axis hyperactivity; Favorable symptoms changes.	Katz et al. (2017)
ABT-436 (V1b antagonist)	Human (alcohol dependence)	Phase 2 in clinical trial for alcohol-dependence; Increase of alcohol abstinence; Reduction of alcohol outcomes for subjects with higher baseline levels of stress.	Ryan et al. (2017)
**GR modulation**			
CORT108297 (sGRm)	3xTg-AD mice	Reduction of APP C-terminal fragments and p25 levels.	Baglietto-Vargas et al. (2013)
	Wistar rats	Attenuation of electroconvulsive shock-induced retrograde amnesia.	Andrade et al. (2012)
	Sprague–Dawley rats	Reduction of neuroendocrine stress responses and immobility in the FST.	Solomon et al. (2014)
	oAβ_25–35_ rat	Reverse oAβ_25–35_-induced neuroinflammatory and apoptotic processes, cognitive and synaptic deficits, and APP misprocessing.	Pineau et al. (2016)
CORT113176 (sGRm)	Wobbler mice	Reduction of neurodegeneration and neuroinflammation.	Meyer et al. (2014)
	oAβ_25–35_ rat	Reverse oAβ_25–35_-induced neuroinflammatory and apoptotic processes, cognitive and synaptic deficits, and APP misprocessing.	Pineau et al. (2016)
**GC synthesis inhibition**			
UE1961 (11β-HSD1 inhibitor)	Aged mice	Improvement of short-term memory.	Sooy et al. (2010)
UE2316 (11β-HSD1 inhibitor)	Tg2576-AD mice	Reduction of Aβ plaques in cortex; Increase of IDE levels; Memory improvements.	Sooy et al. (2015)
A-918446 (11β-HSD1 inhibitor)	Aged rodents	Improvement of memory consolidation and recall in inhibitory avoidance; Increase of CREB phosphorylation.	Mohler et al. (2011)
A-801195 (11β-HSD1 inhibitor)		Improvement of short-term memory	
11β-HSD1 knock-out	Aged mice	Prevention of intra-neuronal corticosterone increase; Improvement of long term memory (watermaze).	Yau et al. (2001)
Metyrapone (1lβ-hydroxylase inhibitor)	SAMP8 mice	Prevention of stress-induced corticosterone elevation, spatial memory deficits and hippocampal neurons loss.	Iinuma et al. (2008)
UE2343 (11β-HSD1 inhibitor)	Human	Phase 1 of clinical trial: compound safe, well tolerated and able to penetrate the brain (healthy subjects).	Webster et al. (2017)
Carbenoxolone (11β-HSD1 inhibitor)	Aging human	Improvement of verbal memory and fluency.	Sandeep et al. (2004)

Such molecules have already been used in clinical trials for mood disorders (Zorrilla and Koob, 2004, 2010) and MDD (Nielsen, 2006) for many years, with disappointing results. However, all these preclinical studies highlight a promising therapeutic potential of CRH-R1 antagonists as treatments for neurodegenerative disorders such as AD. New CRH-R1 antagonists are in development to improve their bioavailability and safety profiles (Spierling and Zorrilla, 2017).

### Targeting AVP Receptors In AD

Hypothalamic neurons of PVN and supra-optic nucleus are the major source of AVP (Swaab, 1998). This hormone is especially involved in the regulation of water, electrolyte balance and in the stress response (Swaab, 1995; Twist et al., 2000). AVP is also implicated in many central processes including learning and memory (de Wied et al., 1993; Caldwell et al., 2008; Lee et al., 2009), anxiety (Caldwell et al., 2008; Lee et al., 2009), and processing of social information (Cilz et al., 2018). The effects of AVP are mediated by different receptor subtypes: V1a, V1b, and V2, which are G-protein coupled receptors (Cilz et al., 2018). However, the limbic action of AVP seems to be mediated by the V1b subtype, enriched in the hippocampus (Young et al., 2006). In rodents, its pharmacological activation enhances excitatory post-synaptic currents (EPSCs) that are absent in V1b^−/−^ mice. V1b potentiation of EPSCs is dependent of N-methyl-D-aspartate (NMDA) receptors activation and intracellular Ca^2+^ signaling (Pagani et al., 2015; Cilz et al., 2018).

In a depressive and/or anxiety context, the therapeutic interest of V1b antagonists has been known for many years (Griebel et al., 2003). This target is particularly studied in pathologies associated with HPA axis dysregulation (Griebel et al., 2003; Katz et al., 2016; Table 1). The first selective and orally active V1b antagonist developed was SSR149415. In rodent models of anxiety and depression, SSR149415 produced a clear-cut anxiolytic-like activity in traumatic stress models (*social defeat paradigm*) and antidepressant-like effects (Griebel et al., 2002). More recently, in the same paradigm, other V1b antagonists (*TASP0390325 and TASP0233278*) elicited antidepressant-like effects (Iijima et al., 2014). These findings evidenced that V1b receptor blockade is particularly interesting for the treatment of affective and emotional disorders. For instance, several clinical trials are in progress with the V1b antagonist ABT-436, which is in phase 1b for depression treatment (Katz et al., 2016, 2017), and in phase 2 for alcohol dependence treatment (Ryan et al., 2017).

Thus, while evidences about a direct involvement of hypothalamic AVP in AD patients are not yet available (Goudsmit et al., 1990; Lucassen et al., 1994; Ishunina et al., 2002), many studies showed a crucial role played by AVP in stress and stress-related disorders such as MDD or anxiety. These findings are interesting as MDD could be a prodromal feature of AD and dementia (Herbert and Lucassen, 2016; Ishijima et al., 2017; Canet et al., 2018), justifying further investigations.

### Targeting GC Synthesis In AD

11β-HSD1 is a key enzyme that mediates the intracellular conversion of inactive cortisone to cortisol in humans (*11β-dehydrocorticosterone to corticosterone in rodent*), the active form of GC, thus amplifying steroid action. An haplotype in the 11β-HSD1 gene that increases 6-fold the risk to develop AD was reported (de Quervain et al., 2004).

Lowering GC exposure in the brain *via* intracellular inhibition of 11β-HSD1 has emerged as a therapeutic strategy to treat cognitive impairment in AD (Table 1). Pharmacological inhibition of 11β-HSD1 (*UE1961*) in aged mice improved spatial memory performance (Sooy et al., 2010). More recently, authors also demonstrated using another inhibitor (*UE2316*), a reduction of Aβ plaques in the cortex of old Tg2576-AD mice associated with an increase of IDE levels and memory improvements (Sooy et al., 2015). Moreover, in aged rodents, modulation of 11β-HSD1 by genetic knockdown or pharmacological inhibition improved memory. In Molher’s study, the authors tested an acute treatment with two novels inhibitors (*A-918446 and A-801195, Abbott Laboratories*). These compounds were able to improve memory consolidation, short-term memory, and recall in inhibitory avoidance (Mohler et al., 2011).

It is also known that aged mice develop elevated plasma corticosterone levels that correlate with learning deficits in the water-maze. This poor performance in a long-term memory task can be ameliorated by an 11β-HSD1 knockout, implicating lower intraneuronal corticosterone levels (Yau et al., 2001, 2015). In senescence-accelerated SAMP8 mice, pre-treatment with metyrapone, which inhibits the 11β-hydroxylase, completely normalized corticosterone levels and restored spatial memory (Iinuma et al., 2008).

These promising data led to a clinical trial with a novel brain-penetrant 11β-HSD1 inhibitor (UE2343, Xanamem^TM^) for AD treatment. The Phase 1 clinical studies showed that UE2343 is safe, well tolerated and is able to moderately penetrate the brain (Webster et al., 2017). In addition, it is interesting to note that treatment of cognitively impaired elderly male patients with carbenoxolone (*a non-selective 11β-HSD1 inhibitor*) improved verbal fluency and memory (Sandeep et al., 2004).

### Targeting GR In AD

The ubiquitous expression of GR and their involvement in a broad range of biological processes make them a prime target in many diseases. On the other hand, the global inhibition or activation of these receptors can lead to many potential side effects. For instance, it was demonstrated that a blockade of brain GR with Mifepristone (RU486, the reference non-selective GR antagonist) impaired spatial learning and memory in rodent (Roozendaal and McGaugh, 1997; Oitzl et al., 1998). A more recent study showed that central or peripheral administration of RU486, immediately following memory reactivation, induced a deficit in post-retrieval long-term memory, and memory did not re-emerge after a footshock reminder (Nikzad et al., 2011). Indeed, GC, GR and associated signaling pathways are crucial for memory consolidation. Acute stress and GR activation were shown to enhance memory consolidation and to inhibit working memory at the same time (Barsegyan et al., 2010). Many factors should be taken into consideration to understand and predict these GC contradictory effects. Depending on the brain area and on acute vs. chronic stress conditions, GC/GR can exert opposite effects (Roozendaal, 2002; McEwen, 2008; Barsegyan et al., 2010; Figure 1).

It is also important to mention the key role of GR in inflammatory processes. GR are necessary to maintain homeostasis since they control the expression of a large part of anti- and pro-inflammatory genes (Barnes, 1998; Van Bogaert et al., 2011; Coutinho and Chapman, 2011). An antagonist can prevent a crucial action of GR and lead to severe side effects associated with aberrant inflammatory processes. For example, it was shown that RU486 blocked the anti-inflammatory effects of exercise in a murine model of allergen-induced pulmonary inflammation (Pastva et al., 2005).

In AD, it was reported that patients treated with prednisone (*a GC used for its anti-inflammatory properties*) presented a more pronounced behavioral decline compared to the placebo-treated cohort (Aisen, 2000). Similarly, it was shown that GC administration (*dexamethasone*) in 3xTg-AD mice increases Aβ pathology and subsequent Tau accumulation and hyperphosphorylation (Green et al., 2006). It results inexorably in a vicious cycle whereby the pathology increases the secretion of GC, which further enhances the pathology (Baglietto-Vargas et al., 2013; Brureau et al., 2013; Pineau et al., 2016; Canet et al., 2018, 2019). All these findings tend to prove that in AD, but also in all pathologies implying GC, both GR agonists or antagonists should be used with caution.

Recently, in a search to abrogate potential side effects of GR antagonism, new compounds were developed and act as highly selective GR modulators (sGRm; Clark et al., 2008; Beaudry et al., 2014; Hunt et al., 2015; Pineau et al., 2016; Meijer et al., 2018; Viho et al., 2019). The use of sGRm is an attractive approach to separate wanted from unwanted outcomes. Different sGRm were developed and vary substantially in their biological activities. These molecules have the ability to act as agonist, as well as antagonist depending on the tissue or gene targets, but also on the physiological context. However, one thing to keep in mind is that the prediction of exact sGRm action is really challenging considering the numerous factors to take into account. Meijer et al. (2018) recently stated about the complexity of sGRm mechanisms: “*even if we know the coactivators that will be recruited by a sGRm-GR complex, in most cases it is unknown which signaling pathways are involved in which transcriptional process. Given the large number of coactivators and their highly gene- and tissue-specific regulation, such analyses are very time consuming, if informative”*.

In a direct application, sGRm offered promising results (Table 1). One of them, and the first to be developed by Corcept Therapeutics (Menlo Park, San Mateo, CA, USA), is CORT108297. It was first described as an antagonist (Belanoff et al., 2010), but its modulatory properties were discovered few years later (Zalachoras et al., 2013). In fact, the authors surprisingly observed that CORT108297 induced a unique interaction profile between GR and its coregulators compared with the full agonist dexamethasone and the non-selective antagonist, RU486. This atypical profile could explain the paradoxical effects of CORT108297 according to the brain region (Zalachoras et al., 2013; Viho et al., 2019). In rats, CORT108297 attenuates electroconvulsive shock-induced retrograde amnesia (Andrade et al., 2012). This molecule also presents antidepressant properties and reduces neuroendocrine stress responses and immobility in the forced-swimming test (FST), alike imipramine, a classic treatment for MDD (Solomon et al., 2014). By contrast, treatment with another member of this family, CORT118335, which has the particularity of being a GR modulator but also a MR antagonist, did not affect immobility in the FST (Nguyen et al., 2018), confirming a differential specificity and efficacy of each molecule.

In the 3xTg-AD mice, a 21 days treatment with CORT108297 reduced APP C-terminal fragments and p25 levels (Baglietto-Vargas et al., 2013), which is the activator of cyclin dependent kinase 5 (Cdk5), involved in AD pathophysiology (Patrick et al., 1999). In an acute rat model of AD (the oAβ_25–35_ model), we tested the therapeutic potential of CORT108297 and CORT113176 (Pineau et al., 2016). CORT113176 is also a sGRm, but displays a better affinity for GR than CORT108297 (Beaudry et al., 2014; Pineau et al., 2016). In this acute model, we previously evidenced a strong, long-lasting activation of the HPA axis, associated with a modification of GR and MR expression in brain regions involved in the control of GC secretion (hippocampus, amygdala and hypothalamus; Brureau et al., 2013). We found that both compounds (CORT113176 at a lower dose than CORT108297) were able to reverse neuroinflammatory and apoptotic processes, cognitive and synaptic deficits, and APP misprocessing (Pineau et al., 2016). To confirm the interest of sGRm, we compared these compounds with RU486. Interestingly, we observed that this non-selective antagonist only partially reversed previously observed pathological impairments (Pineau et al., 2016). In the same line of evidence, CORT113176 showed promising effects in a mouse model of amyotrophic lateral sclerosis by reducing neuronal injury and neuroinflammation in the spinal cord (Meyer et al., 2014). Thus, even if these compounds seemed to exhibit predominantly antagonistic actions in limbic structures in pathological conditions (Andrade et al., 2012; Atucha et al., 2015; Pineau et al., 2016), they could avoid side effects of GR blockade by some agonistic properties.

## Conclusions

If we postulate that AD is a stress-related disorder, targeting HPA axis hormones and receptors could be an attractive approach and pave the way for new therapeutic strategies. Indeed, due to its primordial role in the maintenance of homeostasis, the HPA axis seems to be a key-actor in the etiology of AD and a prime target to tackle the pathology by offering multiple angles of action. Several strategies aiming to restore a normal functionality and activity of the HPA axis are in development with promising results. Some of these molecules are already in clinical studies, not necessarily in an AD context. Since some of them already obtained a safe and tolerated profile, testing them in AD patients could be a really encouraging challenge in the near future. Finally, this review wishes also to alert about the risk in prescribing GC-based therapies in the elderly or in early AD patients.

## Author Contributions

GC, CH, CZ, NC, CD and LG equally contributed to the definition of the scope and to writing of the manuscript.

## Conflict of Interest

The authors declare that the research was conducted in the absence of any commercial or financial relationships that could be construed as a potential conflict of interest.

## References

[B1] Aisen P. S. (2000). Anti-inflammatory therapy for Alzheimer’s disease: implications of the prednisone trial. Acta Neurol. Scand. Suppl. 176, 85–89. 10.1034/j.1600-0404.2000.00312.x 11261810

[B2] Andrade C. Shaikh S. A. Narayan L. Blasey C. Belanoff J. (2012). Administration of a selective glucocorticoid antagonist attenuates electroconvulsive shock-induced retrograde amnesia. J. Neural Transm. 119, 337–344. 10.1007/s00702-011-0712-8 21922193

[B3] Atucha E. Zalachoras I. van den Heuvel J. K. van Weert L. T. C. M. Melchers D. Mol I. M.. (2015). A mixed glucocorticoid/mineralocorticoid selective modulator with dominant antagonism in the male rat brain. Endocrinology 156, 4105–4114. 10.1210/en.2015-1390 26305887

[B4] Baglietto-Vargas D. Medeiros R. Martinez-Coria H. LaFerla F. M. Green K. N. (2013). Mifepristone alters amyloid precursor protein processing to preclude amyloid β and also reduces tau pathology. Biol. Psychiatry 74, 357–366. 10.1016/j.biopsych.2012.12.003 23312564 PMC3633722

[B5] Bale T. L. Vale W. W. (2004). CRF and CRF receptors: role in stress responsivity and other behaviors. Annu. Rev. Pharmacol. Toxicol. 44, 525–557. 10.1146/annurev.pharmtox.44.101802.121410 14744257

[B6] Barnes P. J. (1998). Anti-inflammatory actions of glucocorticoids: molecular mechanisms. Clin. Sci. 94, 557–572. 10.1042/cs0940557 9854452

[B7] Barsegyan A. Mackenzie S. M. Kurose B. D. McGaugh J. L. Roozendaal B. (2010). Glucocorticoids in the prefrontal cortex enhance memory consolidation and impair working memory by a common neural mechanism. Proc. Natl. Acad. Sci. U S A 107, 16655–16660. 10.1073/pnas.1011975107 20810923 PMC2944727

[B8] Beaudry J. L. Dunford E. C. Teich T. Zaharieva D. Hunt H. Belanoff J. K.. (2014). Effects of selective and non-selective glucocorticoid receptor: II antagonists on rapid-onset diabetes in young rats. PLoS One 9:e91248. 10.1371/journal.pone.0091248 24642683 PMC3958344

[B9] Behan D. P. Heinrichs S. C. Troncoso J. C. Liu X. J. Kawas C. H. Ling N.. (1995). Displacement of corticotropin releasing factor from its binding protein as a possible treatment for Alzheimer’s disease. Nature 378, 284–287. 10.1038/378284a0 7477348

[B10] Belanoff J. K. Blasey C. M. Clark R. D. Roe R. L. (2010). Selective glucocorticoid receptor (type II) antagonist prevents and reverses olanzapine-induced weight gain. Diabetes Obes. Metab. 12, 545–547. 10.1111/j.1463-1326.2009.01185.x 20518810

[B11] Bengoetxea X. de Cerain A. L. Azqueta A. Ramirez M. J. (2016). Purported interactions of amyloid-β and glucocorticoids in cytotoxicity and genotoxicity: implications in Alzheimer’s disease. J. Alzheimers Dis. 54, 1085–1094. 10.3233/jad-160636 27589535

[B12] Blennow K. de Leon M. J. Zetterberg H. (2006). Alzheimer’s disease. Lancet 368, 387–403. 10.1016/S0140-6736(06)69113-7 16876668

[B14] Brureau A. Zussy C. Delair B. Ogier C. Ixart G. Maurice T.. (2013). Deregulation of hypothalamic-pituitary-adrenal axis functions in an Alzheimer’s disease rat model. Neurobiol. Aging 34, 1426–1439. 10.1016/j.neurobiolaging.2012.11.015 23273603

[B15] Buijs R. M. (1990). Vasopressin and oxytocin localization and putative functions in the brain. Acta Neurochir. Suppl. 47, 86–89. 10.1007/978-3-7091-9062-3_10 2407060

[B16] Caldwell H. K. Lee H.-J. Macbeth A. H. Young W. S. III. (2008). Vasopressin: behavioral roles of an “original” neuropeptide. Prog. Neurobiol. 84, 1–24. 10.1016/j.pneurobio.2007.10.007 18053631 PMC2292122

[B17] Campbell S. N. Zhang C. Monte L. Roe A. D. Rice K. C. Taché Y.. (2014). Increased tau phosphorylation and aggregation in the hippocampus of mice overexpressing corticotropin-releasing factor. J. Alzheimers Dis. 43, 967–976. 10.3233/jad-141281 25125464 PMC4258165

[B18] Canet G. Chevallier N. Perrier V. Desrumaux C. Givalois L. (2019). “Targeting glucocorticoid receptors: a new avenue for Alzheimer’s disease therapy,” in Pathology, Prevention and Therapeutics of Neurodegenerative Disease, eds Singh S. Joshi N. (Singapore: Springer Singapore), 173–183.

[B19] Canet G. Chevallier N. Zussy C. Desrumaux C. Givalois L. (2018). Central role of glucocorticoid receptors in Alzheimer’s disease and depression. Front. Neurosci. 12:739. 10.3389/fnins.2018.00739 30459541 PMC6232776

[B20] Carroll J. C. Iba M. Bangasser D. A. Valentino R. J. James M. J. Brunden K. R.. (2011). Chronic stress exacerbates tau pathology, neurodegeneration, and cognitive performance through a corticotropin-releasing factor receptor-dependent mechanism in a transgenic mouse model of tauopathy. J. Neurosci. 31, 14436–14449. 10.1523/JNEUROSCI.3836-11.2011 21976528 PMC3230070

[B21] Chrousos G. P. Detera-Wadleigh S. D. Karl M. (1993). Syndromes of glucocorticoid resistance. Ann. Intern. Med. 119, 1113–1124. 10.7326/0003-4819-119-11-199312010-00009 8239231

[B22] Cilz N. I. Cymerblit-Sabba A. Young W. S. (2018). Oxytocin and vasopressin in the rodent hippocampus. Genes Brain Behav. 18:e12535. 10.1111/gbb.12535 30378258

[B23] Clark R. D. Ray N. C. Williams K. Blaney P. Ward S. Crackett P. H.. (2008). 1H-Pyrazolo[3,4-g]hexahydro-isoquinolines as selective glucocorticoid receptor antagonists with high functional activity. Bioorg. Med. Chem. Lett. 18, 1312–1317. 10.1016/j.bmcl.2008.01.027 18226897

[B24] Coutinho A. E. Chapman K. E. (2011). The anti-inflammatory and immunosuppressive effects of glucocorticoids, recent developments and mechanistic insights. Mol. Cell. Endocrinol. 335, 2–13. 10.1016/j.mce.2010.04.005 20398732 PMC3047790

[B25] Csernansky J. G. Dong H. Fagan A. M. Wang L. Xiong C. Holtzman D. M.. (2006). Plasma cortisol and progression of dementia in subjects with Alzheimer-type dementia. Am. J. Psychiatry 163, 2164–2169. 10.1176/appi.ajp.163.12.2164 17151169 PMC1780275

[B26] de Quervain D. J.-F. Poirier R. Wollmer M. A. Grimaldi L. M. E. Tsolaki M. Streffer J. R.. (2004). Glucocorticoid-related genetic susceptibility for Alzheimer’s disease. Hum. Mol. Genet. 13, 47–52. 10.1093/hmg/ddg361 14583441

[B27] de Wied D. Diamant M. Fodor M. (1993). Central nervous system effects of the neurohypophyseal hormones and related peptides. Front. Neuroendocrinol. 14, 251–302. 10.1006/frne.1993.1009 8258377

[B28] Dong H. Keegan J. M. Hong E. Gallardo C. Montalvo-Ortiz J. Wang B.. (2018). Corticotrophin releasing factor receptor 1 antagonists prevent chronic stress-induced behavioral changes and synapse loss in aged rats. Psychoneuroendocrinology 90, 92–101. 10.1016/j.psyneuen.2018.02.013 29477954 PMC5864558

[B29] Dong H. Wang S. Zeng Z. Li F. Montalvo-Ortiz J. Tucker C.. (2014). Effects of corticotrophin-releasing factor receptor 1 antagonists on amyloid-β and behavior in Tg2576 mice. Psychopharmacology 231, 4711–4722. 10.1007/s00213-014-3629-8 24862368 PMC4233002

[B30] Dong H. Yuede C. M. Yoo H.-S. Martin M. V. Deal C. Mace A. G.. (2008). Corticosterone and related receptor expression are associated with increased β-amyloid plaques in isolated Tg2576 mice. Neuroscience 155, 154–163. 10.1016/j.neuroscience.2008.05.017 18571864 PMC2664643

[B31] Givalois L. (2014). The glucocorticoid receptors regulation in Alzheimer’s disease. Neurobiol. Aging 35, e17–e18. 10.1016/j.neurobiolaging.2013.12.012 24411291

[B32] Goudsmit E. Hofman M. A. Fliers E. Swaab D. F. (1990). The supraoptic and paraventricular nuclei of the human hypothalamus in relation to sex, age and Alzheimer’s disease. Neurobiol. Aging 11, 529–536. 10.1016/0197-4580(90)90114-f 2234284

[B33] Green K. N. Billings L. M. Roozendaal B. McGaugh J. L. LaFerla F. M. (2006). Glucocorticoids increase amyloid-β and tau pathology in a mouse model of Alzheimer’s disease. J. Neurosci. 26, 9047–9056. 10.1523/JNEUROSCI.2797-06.2006 16943563 PMC6675335

[B34] Griebel G. Simiand J. Serradeil-Le Gal C. Wagnon J. Pascal M. Scatton B.. (2002). Anxiolytic- and antidepressant-like effects of the non-peptide vasopressin V1b receptor antagonist, SSR149415, suggest an innovative approach for the treatment of stress-related disorders. Proc. Natl. Acad. Sci. U S A 99, 6370–6375. 10.1073/pnas.092012099 11959912 PMC122955

[B35] Griebel G. Simiand J. Stemmelin J. Gal C. S.-L. Steinberg R. (2003). The vasopressin V1b receptor as a therapeutic target in stress-related disorders. Curr. Drug Targets CNS Neurol. Disord. 2, 191–200. 10.2174/1568007033482850 12769799

[B36] Hartmann A. Veldhuis J. D. Deuschle M. Standhardt H. Heuser I. (1997). Twenty-four hour cortisol release profiles in patients with Alzheimer’s and Parkinson’s disease compared to normal controls: ultradian secretory pulsatility and diurnal variation. Neurobiol. Aging 18, 285–289. 10.1016/s0197-4580(97)80309-0 9263193

[B37] Heininger K. (2000). A unifying hypothesis of Alzheimer’s disease. IV. Causation and sequence of events. Rev. Neurosci. 11, 213–328. 10.1515/REVNEURO.2000.11.S1.213 11065271

[B38] Herbert J. Lucassen P. J. (2016). Depression as a risk factor for Alzheimer’s disease: genes, steroids, cytokines and neurogenesis—what do we need to know? Front. Neuroendocrinol. 41, 153–171. 10.1016/j.yfrne.2015.12.001 26746105

[B39] Hunt H. J. Belanoff J. K. Golding E. Gourdet B. Phillips T. Swift D.. (2015). 1H-Pyrazolo[3,4-g]hexahydro-isoquinolines as potent GR antagonists with reduced hERG inhibition and an improved pharmacokinetic profile. Bioorg. Med. Chem. Lett. 25, 5720–5725. 10.1016/j.bmcl.2015.10.097 26546213

[B40] Iijima M. Yoshimizu T. Shimazaki T. Tokugawa K. Fukumoto K. Kurosu S.. (2014). Antidepressant and anxiolytic profiles of newly synthesized arginine vasopressin V_1B_ receptor antagonists: TASP0233278 and TASP0390325: pharmacological profiles of V_1B_ receptor antagonists. Br. J. Pharmacol. 171, 3511–3525. 10.1111/bph.12699 24654684 PMC4105937

[B41] Iinuma M. Ichihashi Y. Hioki Y. Kurata C. Tamura Y. Kubo K. (2008). Malocclusion induces chronic stress. Okajimas Folia Anat. Jpn. 85, 35–42. 10.2535/ofaj.85.35 18833910

[B42] Ishijima S. Baba H. Maeshima H. Shimano T. Inoue M. Suzuki T.. (2017). Glucocorticoid may influence amyloid β metabolism in patients with depression. Psychiatry Res. 259, 191–196. 10.1016/j.psychres.2017.10.008 29073556

[B43] Ishunina T. A. Wouda J. Fisser B. Swaab D. F. (2002). Sex differences in estrogen receptor α and β expression in vasopressin neurons of the supraoptic nucleus in elderly and Alzheimer’s disease patients: no relationship with cytoskeletal alterations. Brain Res. 951, 322–329. 10.1016/s0006-8993(02)03269-9 12270512

[B44] Katz D. A. Liu W. Locke C. Dutta S. Tracy K. A. (2016). Clinical safety and hypothalamic-pituitary-adrenal axis effects of the arginine vasopressin type 1B receptor antagonist ABT-436. Psychopharmacology 233, 71–81. 10.1007/s00213-015-4089-5 26407603

[B45] Katz D. A. Locke C. Greco N. Liu W. Tracy K. A. (2017). Hypothalamic-pituitary-adrenal axis and depression symptom effects of an arginine vasopressin type 1B receptor antagonist in a one-week randomized Phase 1b trial. Brain Behav. 7:e00628. 10.1002/brb3.628 28293470 PMC5346517

[B46] Kroon J. Koorneef L. L. van den Heuvel J. K. Verzijl C. R. C. van de Velde N. M. Mol I. M.. (2018). Selective glucocorticoid receptor antagonist CORT125281 activates brown adipose tissue and alters lipid distribution in male mice. Endocrinology 159, 535–546. 10.1210/en.2017-00512 28938459

[B47] Lee H.-J. Macbeth A. H. Pagani J. Young W. S. (2009). Oxytocin: the great facilitator of life. Prog. Neurobiol. 88, 127–151. 10.1016/j.pneurobio.2009.04.001 19482229 PMC2689929

[B48] Lucassen P. J. Salehi A. Pool C. W. Gonatas N. K. Swaab D. F. (1994). Activation of vasopressin neurons in aging and Alzheimer’s disease. J. Neuroendocrinol. 6, 673–679. 10.1111/j.1365-2826.1994.tb00634.x 7894470

[B49] Lupien S. J. de Leon M. de Santi S. Convit A. Tarshish C. Nair N. P.. (1998). Cortisol levels during human aging predict hippocampal atrophy and memory deficits. Nat. Neurosci. 1, 69–73. 10.1038/271 10195112

[B50] Maslov L. N. Naryzhnaya N. V. Boshchenko A. A. Popov S. V. Ivanov V. V. Oeltgen P. R. (2019). Is oxidative stress of adipocytes a cause or a consequence of the metabolic syndrome? J. Clin. Transl. Endocrinol. 15, 1–5. 10.1016/j.jcte.2018.11.001 30479968 PMC6240632

[B51] McEwen B. S. (2008). Central effects of stress hormones in health and disease: understanding the protective and damaging effects of stress and stress mediators. Eur. J. Pharmacol. 583, 174–185. 10.1016/j.ejphar.2007.11.071 18282566 PMC2474765

[B52] Meijer O. C. Koorneef L. L. Kroon J. (2018). Glucocorticoid receptor modulators. Ann. Endocrinol. 79, 107–111. 10.1016/j.ando.2018.03.004 29731108

[B53] Meyer M. Gonzalez Deniselle M. C. Hunt H. de Kloet E. R. De Nicola A. F. (2014). The selective glucocorticoid receptor modulator CORT108297 restores faulty hippocampal parameters in Wobbler and corticosterone-treated mice. J. Steroid Biochem. Mol. Biol. 143, 40–48. 10.1016/j.jsbmb.2014.02.007 24565565

[B54] Mohler E. G. Browman K. E. Roderwald V. A. Cronin E. A. Markosyan S. Scott Bitner R.. (2011). Acute inhibition of 11 -hydroxysteroid dehydrogenase type-1 improves memory in rodent models of cognition. J. Neurosci. 31, 5406–5413. 10.1523/JNEUROSCI.4046-10.2011 21471376 PMC6622712

[B55] Mouri T. Itoi K. Takahashi K. Suda T. Murakami O. Yoshinaga K.. (1993). Colocalization of corticotropin-releasing factor and vasopressin in the paraventricular nucleus of the human hypothalamus. Neuroendocrinology 57, 34–39. 10.1210/me.7.10.1357 8479614

[B56] Nguyen E. T. Caldwell J. L. Streicher J. Ghisays V. Balmer N. J. Estrada C. M.. (2018). Differential effects of imipramine and CORT118335 (Glucocorticoid receptor modulator/mineralocorticoid receptor antagonist) on brain-endocrine stress responses and depression-like behavior in female rats. Behav. Brain Res. 336, 99–110. 10.1016/j.bbr.2017.08.045 28866130

[B57] Nielsen D. M. (2006). Corticotropin-releasing factor type-1 receptor antagonists: the next class of antidepressants? Life Sci. 78, 909–919. 10.1016/j.lfs.2005.06.003 16122764

[B58] Nikzad S. Vafaei A. A. Rashidy-Pour A. Haghighi S. (2011). Systemic and intrahippocampal administrations of the glucocorticoid receptor antagonist RU38486 impairs fear memory reconsolidation in rats. Stress 14, 459–464. 10.3109/10253890.2010.548171 21438774

[B59] Notarianni E. (2013). Hypercortisolemia and glucocorticoid receptor-signaling insufficiency in Alzheimer’s disease initiation and development. Curr. Alzheimer Res. 10, 714–731. 10.2174/15672050113109990137 23906001

[B60] Oitzl M. S. Fluttert M. Sutanto W. de Kloet E. R. (1998). Continuous blockade of brain glucocorticoid receptors facilitates spatial learning and memory in rats. Eur. J. Neurosci. 10, 3759–3766. 10.1046/j.1460-9568.1998.00381.x 9875354

[B61] Pagani J. H. Zhao M. Cui Z. Williams Avram S. K. Caruana D. A. Dudek S. M.. (2015). Role of the vasopressin 1b receptor in rodent aggressive behavior and synaptic plasticity in hippocampal area CA2. Mol. Psychiatry 20, 490–499. 10.1038/mp.2014.47 24863146 PMC4562468

[B63] Pastva A. Estell K. Schoeb T. R. Schwiebert L. M. (2005). RU486 blocks the anti-inflammatory effects of exercise in a murine model of allergen-induced pulmonary inflammation. Brain Behav. Immun. 19, 413–422. 10.1016/j.bbi.2005.04.004 15922554 PMC2891236

[B64] Patrick G. N. Zukerberg L. Nikolic M. de la Monte S. Dikkes P. Tsai L.-H. (1999). Conversion of p35 to p25 deregulates Cdk5 activity and promotes neurodegeneration. Nature 402, 615–622. 10.1038/45159 10604467

[B65] Pineau F. Canet G. Desrumaux C. Hunt H. Chevallier N. Ollivier M.. (2016). New selective glucocorticoid receptor modulators reverse amyloid-β peptide-induced hippocampus toxicity. Neurobiol. Aging 45, 109–122. 10.1016/j.neurobiolaging.2016.05.018 27459932

[B66] Pomara N. Singh R. R. Deptula D. LeWitt P. A. Bissette G. Stanley M.. (1989). CSF corticotropin-releasing factor (CRF) in Alzheimer’s disease: its relationship to severity of dementia and monoamine metabolites. Biol. Psychiatry 26, 500–504. 10.1016/0006-3223(89)90071-1 2477071

[B68] Querfurth H. W. LaFerla F. M. (2010). Alzheimer’s disease. N. Engl. J. Med. 362, 329–344. 10.1056/NEJMra0909142 20107219

[B69] Raff H. (1993). Interactions between neurohypophysial hormones and the ACTH-adrenocortical axis. Ann. N Y Acad. Sci. 689, 411–425. 10.1111/j.1749-6632.1993.tb55564.x 8396873

[B70] Reul J. M. de Kloet E. R. (1985). Two receptor systems for corticosterone in rat brain: microdistribution and differential occupation. Endocrinology 117, 2505–2511. 10.1210/endo-117-6-2505 2998738

[B71] Roozendaal B. (2000). 1999 Curt P. Richter award. Glucocorticoids and the regulation of memory consolidation. Psychoneuroendocrinology 25, 213–238. 10.1016/s0306-4530(99)00058-x 10737694

[B72] Roozendaal B. (2002). Stress and memory: opposing effects of glucocorticoids on memory consolidation and memory retrieval. Neurobiol. Learn. Mem. 78, 578–595. 10.1006/nlme.2002.4080 12559837

[B73] Roozendaal B. McGaugh J. L. (1997). Glucocorticoid receptor agonist and antagonist administration into the basolateral but not central amygdala modulates memory storage. Neurobiol. Learn. Mem. 67, 176–179. 10.1006/nlme.1996.3765 9075247

[B74] Ryan M. L. Falk D. E. Fertig J. B. Rendenbach-Mueller B. Katz D. A. Tracy K. A.. (2017). A phase 2, double-blind, placebo-controlled randomized trial assessing the efficacy of ABT-436, a novel V1b receptor antagonist, for alcohol dependence. Neuropsychopharmacology 42, 1012–1023. 10.1038/npp.2016.214 27658483 PMC5506792

[B75] Sandeep T. C. Yau J. L. W. MacLullich A. M. J. Noble J. Deary I. J. Walker B. R.. (2004). 11β-hydroxysteroid dehydrogenase inhibition improves cognitive function in healthy elderly men and type 2 diabetics. Proc. Natl. Acad. Sci. U S A 101, 6734–6739. 10.1073/pnas.0306996101 15071189 PMC404114

[B76] Sapolsky R. M. (1996). Stress, glucocorticoids, and damage to the nervous system: the current state of confusion. Stress 1, 1–19. 10.3109/10253899609001092 9807058

[B77] Solomon M. B. Wulsin A. C. Rice T. Wick D. Myers B. McKlveen J.. (2014). The selective glucocorticoid receptor antagonist CORT 108297 decreases neuroendocrine stress responses and immobility in the forced swim test. Horm. Behav. 65, 363–371. 10.1016/j.yhbeh.2014.02.002 24530653 PMC4074011

[B78] Sooy K. Noble J. McBride A. Binnie M. Yau J. L. W. Seckl J. R.. (2015). Cognitive and disease-modifying effects of 11β-hydroxysteroid dehydrogenase type 1 inhibition in male Tg2576 mice, a model of Alzheimer’s disease. Endocrinology 156, 4592–4603. 10.1210/en.2015-1395 26305888 PMC4655221

[B79] Sooy K. Webster S. P. Noble J. Binnie M. Walker B. R. Seckl J. R.. (2010). Partial deficiency or short-term inhibition of 11 -hydroxysteroid dehydrogenase type 1 improves cognitive function in aging mice. J. Neurosci. 30, 13867–13872. 10.1523/JNEUROSCI.2783-10.2010 20943927 PMC3016616

[B80] Sotiropoulos I. Sousa N. (2016). Tau as the converging protein between chronic stress and Alzheimer’s disease synaptic pathology. Neurodegener. Dis. 16, 22–25. 10.1159/000440844 26551025

[B81] Spierling S. R. Zorrilla E. P. (2017). Don’t stress about CRF: assessing the translational failures of CRF1antagonists. Psychopharmacology 234, 1467–1481. 10.1007/s00213-017-4556-2 28265716 PMC5420464

[B83] Swaab D. F. (1995). Ageing of the human hypothalamus. Horm. Res. 43, 8–11. 10.1159/000184230 7721267

[B84] Swaab D. F. (1998). The human hypothalamo-neurohypophysial system in health and disease. Prog. Brain Res. 119, 577–618. 10.1016/s0079-6123(08)61594-0 10074813

[B85] Swanwick G. R. Kirby M. Bruce I. Buggy F. Coen R. F. Coakley D.. (1998). Hypothalamic-pituitary-adrenal axis dysfunction in Alzheimer’s disease: lack of association between longitudinal and cross-sectional findings. Am. J. Psychiatry 155, 286–289. 10.1176/ajp.155.2.286 9464214

[B86] Tasker J. G. Herman J. P. (2011). Mechanisms of rapid glucocorticoid feedback inhibition of the hypothalamic-pituitary-adrenal axis. Stress 14, 398–406. 10.3109/10253890.2011.586446 21663538 PMC4675656

[B87] Thomas S. A. (2015). Neuromodulatory signaling in hippocampus-dependent memory retrieval. Hippocampus 25, 415–431. 10.1002/hipo.22394 25475876 PMC9484472

[B88] Tilders F. J. Schmidt E. D. de Goeij D. C. (1993). Phenotypic plasticity of CRF neurons during stress. Ann. N Y Acad. Sci. 697, 39–52. 10.1111/j.1749-6632.1993.tb49921.x 8257021

[B90] Torner L. Plotsky P. M. Neumann I. D. de Jong T. R. (2017). Forced swimming-induced oxytocin release into blood and brain: effects of adrenalectomy and corticosterone treatment. Psychoneuroendocrinology 77, 165–174. 10.1016/j.psyneuen.2016.12.006 28064086

[B91] Twist S. J. Taylor G. A. Weddell A. Weightman D. R. Edwardson J. A. Morris C. M. (2000). Brain oestradiol and testosterone levels in Alzheimer’s disease. Neurosci. Lett. 286, 1–4. 10.1016/s0304-3940(00)01078-8 10822138

[B13] Van Bogaert T. Vandevyver S. Dejager L. Hauwermeiren F. V. Pinheiro I. Petta I.. (2011). Tumor necrosis factor inhibits glucocorticoid receptor function in mice. J. Biol. Chem. 286, 26555–26567. 10.1074/jbc.M110.212365 21646349 PMC3143620

[B92] Viho E. Buurstede J. C. Mahfouz A. Koorneef L. L. van Weert L. T. C. M. Houtman R.. (2019). Corticosteroid action in the brain: the potential of selective receptor modulation. Neuroendocrinology 109, 266–276. 10.1159/000499659 30884490 PMC6878852

[B93] Vitellius G. Trabado S. Bouligand J. Delemer B. Lombès M. (2018). Pathophysiology of glucocorticoid signaling. Ann. Endocrinol. 79, 98–106. 10.1016/j.ando.2018.03.001 29685454

[B94] Vyas A. Bernal S. Chattarji S. (2003). Effects of chronic stress on dendritic arborization in the central and extended amygdala. Brain Res. 965, 290–294. 10.1016/s0006-8993(02)04162-8 12591150

[B95] Vyas A. Pillai A. G. Chattarji S. (2004). Recovery after chronic stress fails to reverse amygdaloid neuronal hypertrophy and enhanced anxiety-like behavior. Neuroscience 128, 667–673. 10.1016/j.neuroscience.2004.07.013 15464275

[B96] Vyas S. Rodrigues A. J. Silva J. M. Tronche F. Almeida O. F. X. Sousa N.. (2016). Chronic stress and glucocorticoids: from neuronal plasticity to neurodegeneration. Neural Plast. 2016:6391686. 10.1155/2016/6391686 27034847 PMC4806285

[B97] Webster S. P. McBride A. Binnie M. Sooy K. Seckl J. R. Andrew R.. (2017). Selection and early clinical evaluation of the brain-penetrant 11β-hydroxysteroid dehydrogenase type 1 (11β-HSD1) inhibitor UE2343 (Xanamem^TM^). Br. J. Pharmacol. 174, 396–408. 10.1111/bph.13699 28012176 PMC5301048

[B98] Whitehouse P. J. Vale W. W. Zweig R. M. Singer H. S. Mayeux R. Kuhar M. J.. (1987). Reductions in corticotropin releasing factor-like immunoreactivity in cerebral cortex in Alzheimer’s disease, Parkinson’s disease, and progressive supranuclear palsy. Neurology 37, 905–909. 10.1212/wnl.37.6.905 3495748

[B99] Whitnall M. H. Smyth D. Gainer H. (1987). Vasopressin coexists in half of the corticotropin-releasing factor axons present in the external zone of the median eminence in normal rats. Neuroendocrinology 45, 420–424. 10.1159/000124768 3295576

[B100] Wu Q. Yang X. Zhang Y. Zhang L. Feng L. (2016). Chronic mild stress accelerates the progression of Parkinson’s disease in A53T α-synuclein transgenic mice. Exp. Neurol. 285, 61–71. 10.1016/j.expneurol.2016.09.004 27637804

[B101] Yau J. L. W. Noble J. Kenyon C. J. Hibberd C. Kotelevtsev Y. Mullins J. J.. (2001). Lack of tissue glucocorticoid reactivation in 11-hydroxysteroid dehydrogenase type 1 knockout mice ameliorates age-related learning impairments. Proc. Natl. Acad. Sci. U S A 98, 4716–4721. 10.1073/pnas.071562698 11274359 PMC31900

[B200] Yau J. L. W. Wheelan N. Noble J. Walker B. R. Webster S. P. Kenyon C. J.. (2015). Intrahippocampal glucocorticoids generated by β-HSD1 affect memory in aged mice. Neurobiol. Aging 36, 334–343. 10.1016/j.neurobiolaging.2014.07.007 25109766 PMC4706164

[B102] Young W. S. Li J. Wersinger S. R. Palkovits M. (2006). The vasopressin 1b receptor is prominent in the hippocampal area CA2 where it is unaffected by restraint stress or adrenalectomy. Neuroscience 143, 1031–1039. 10.1016/j.neuroscience.2006.08.040 17027167 PMC1748954

[B103] Zalachoras I. Houtman R. Atucha E. Devos R. Tijssen A. M. I. Hu P.. (2013). Differential targeting of brain stress circuits with a selective glucocorticoid receptor modulator. Proc. Natl. Acad. Sci. U S A 110, 7910–7915. 10.1073/pnas.1219411110 23613579 PMC3651427

[B104] Zhang C. Kuo C.-C. Moghadam S. H. Monte L. Campbell S. N. Rice K. C.. (2016). Corticotropin-releasing factor receptor-1 antagonism mitigates β amyloid pathology and cognitive and synaptic deficits in a mouse model of Alzheimer’s disease. Alzheimers Dement. 12, 527–537. 10.1016/j.jalz.2015.09.007 26555315 PMC4860182

[B105] Zorrilla E. P. Koob G. F. (2004). The therapeutic potential of CRF1 antagonists for anxiety. Expert Opin. Investig. Drugs 13, 799–828. 10.1517/13543784.13.7.799 15212620

[B106] Zorrilla E. P. Koob G. F. (2010). Progress in corticotropin-releasing factor-1 antagonist development. Drug Discov. Today 15, 371–383. 10.1016/j.drudis.2010.02.011 20206287 PMC2864802

